# Supplementary material for: Independent tuning of size and coverage of supported Pt nanoparticles using atomic layer deposition
Source: Nat Commun. 2017 Oct 20;8:1074. doi: 10.1038/s41467-017-01140-z (PMC5651928; doi:10.1038/s41467-017-01140-z)
Supplement: Supplementary file 1 — Supplementary Information [file 41467_2017_1140_MOESM1_ESM.pdf]

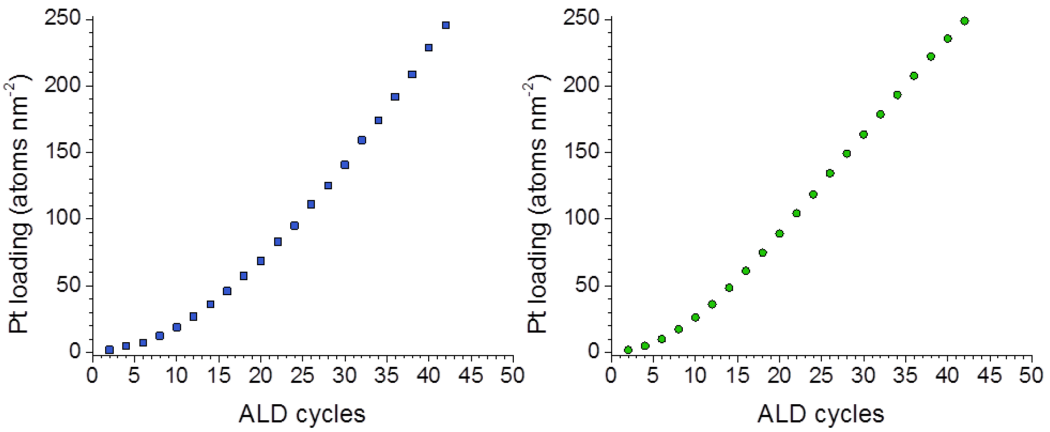

Supplementary Figure 1 | *In situ* elemental characterization during Pt ALD. Pt loading, extracted from RBS-calibrated *in situ* XRF measurements, against the number of deposited ALD cycles for O_2-based Pt ALD (left) and N_2^*-based Pt ALD (right).

Supplementary Note 1: GISAXS analysis strategy

Analysis of the experimental 2D GISAXS patterns is done via a three-step approach (Supplementary Fig. 2). In a first step, the Pt nanoparticles are modeled as full spheroids without size distribution arranged on a regular 2D lattice and composed of the amount of Pt deposited on the unit cell surface area (as determined by XRF). Based on this simplified geometrical model[1-3] and "fast analysis" of one-dimensional (1D) line profiles of the GISAXS patterns, initial estimates for the average particle center-to-center distance $\langle D \rangle$, particle height $\langle H \rangle$ and particle width $\langle W \rangle$ are obtained. The second analysis step uses these values as input parameters for a more sophisticated model of the particle shape, but still without size distribution, to calculate horizontal and vertical line profiles using the software IsGISAXS.[4] The calculated profiles are then compared to the experimental line profiles and the values for $\langle D \rangle$, $\langle H \rangle$ and $\langle W \rangle$ are further optimized to reproduce well the positions of the intensity maxima and minima in the profiles. Third, these optimized values are used as fixed input parameters for a model that now also takes into account a particle size distribution. The 2D GISAXS patterns calculated based on this model show good agreement with the experimental GISAXS patterns, confirming the validity of the analysis approach in step 1 and 2 to extract values for $\langle D \rangle$, $\langle H \rangle$ and $\langle W \rangle$. The analysis approach is schematically summarized in Supplementary Fig. 2 and elaborated in more detail in the following paragraphs.

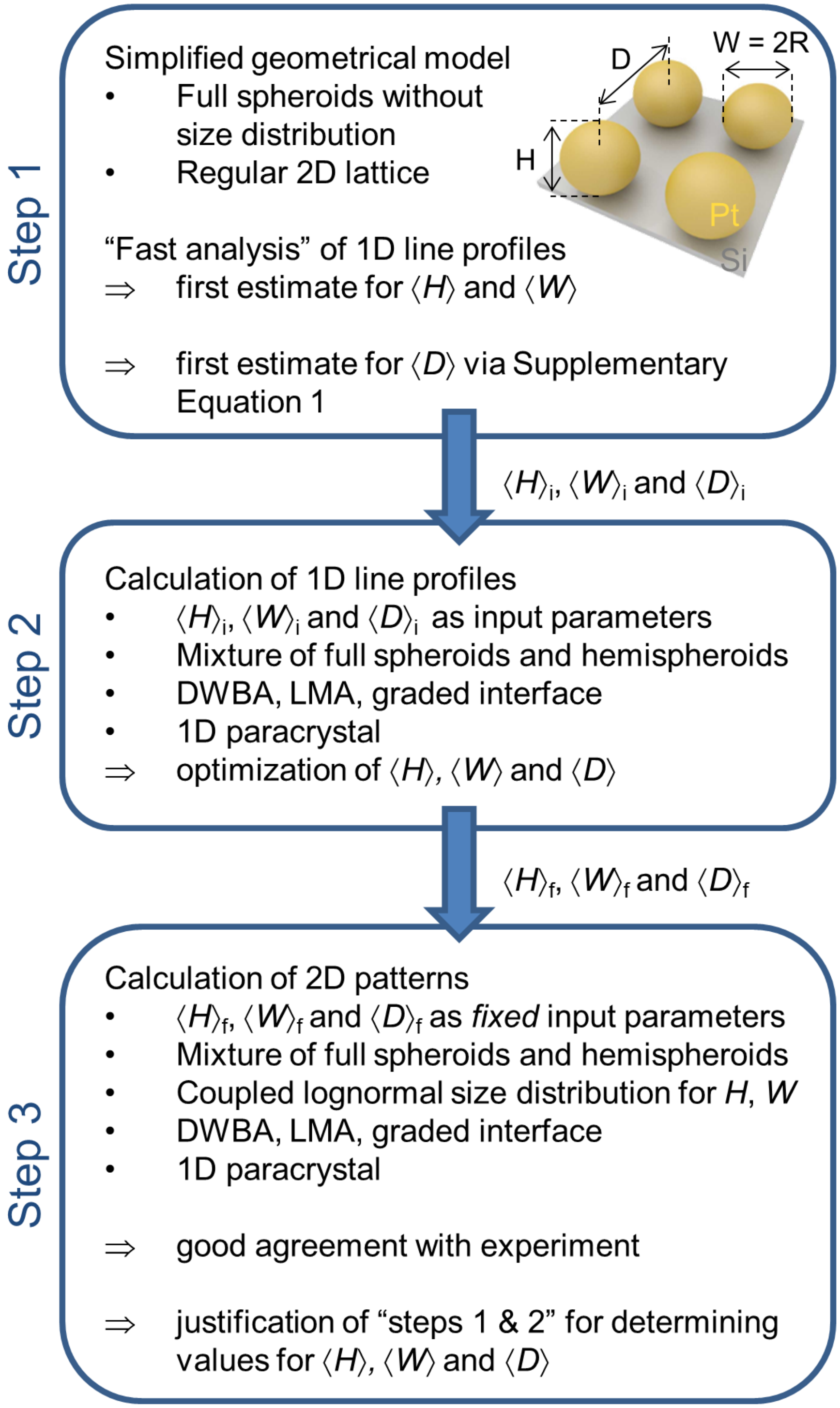

Supplementary Figure 2 | GISAXS analysis strategy. Chart describing the three steps in the GISAXS analysis approach.

Step 1

Initial estimates for the average particle height $\langle H\rangle$ and width $\langle W\rangle$ are obtained via "fast analysis" of 1D line profiles. The average particle height $\langle H\rangle$ is estimated as $2\pi/\Delta q_z$, where Δq_z is the average distance between adjacent minima observed in a line profile taken in the vertical direction, along q_z, at the q_y-position of maximum intensity. The average particle width $\langle W\rangle$ is estimated as $4.4/q_{y,min}$,[5] with $q_{y,min}$ the position of the minimum observed in a line profile taken in the horizontal direction at the Si Yoneda position, i.e. at $q_z = 0.722\ nm^{-1}$. The mean distance parameter $\langle D\rangle$ is then calculated based on the assumed particle shape (full spheroids), the estimated particle dimensions ($\langle H\rangle$ and $\langle W\rangle$), and known Pt loading:

$$\langle D\rangle = \sqrt{\frac{\langle V_{spheroid}\rangle \cdot 66.24 \, {}^{atoms}/_{nm^3}}{S_{Pt}}} = \sqrt{\frac{\left(\frac{2}{3}\pi\langle R\rangle^2\langle H\rangle\right)\cdot 66.24 \, {}^{atoms}/_{nm^3}}{S_{Pt}}} \quad (1)$$

with $\langle V_{spheroid}\rangle = \frac{2}{3}\pi\langle R\rangle^2\langle H\rangle$ the average volume of one Pt nanoparticle (in nm^3), 66.24 atoms nm^{-3} the bulk density of Pt, and S_{Pt} the surface density of Pt atoms as determined by XRF in atoms nm^{-2} (see Supplementary Fig. 1).

Step 2

To further optimize the values for $\langle D\rangle$, $\langle H\rangle$ and $\langle W\rangle$, 1D horizontal and vertical line profiles are calculated using the software IsGISAXS.[4] To this end, a particle shape has to be selected. Based on the tomography result presented in Figure 2 and because the arc-like scattering features in the experimental 2D GISAXS patterns suggest a round shape of the Pt particles, spheroidal nanoclusters are assumed. As will be further explained in the next section, best agreement with the experimental GISAXS patterns is obtained when assuming a two particle model consisting of 50% (75%) full spheroids and 50% (25%) hemi-spheroids for the Pt particles deposited with the O_2-(N_2^*-)based ALD process. Both particle types are assumed to have the same average particle height $\langle H\rangle$ and width $\langle W\rangle$. Therefore, both these particle shapes yield the same volume per Pt cluster so that Supplementary Equation 1 can still be applied to ensure that the amount of Pt per surface area, as simulated in GISAXS, corresponds to the experimentally determined value by XRF. For form factor calculation, the distorted-wave Born approximation (DWBA) is used, including the model of the graded interface used to describe the perturbation caused by densely

packed particles on a surface.[6] The interference function is calculated based on the 1D paracrystal model, which is a regular 1D lattice with a loss of long-range order. The width of the main scattering peak in the horizontal line profile is reproduced well when the Gaussian disorder parameter ω for the center-to-center distance D distribution is set to a value of ~$0.4\cdot\langle D\rangle$.

The values for $\langle D\rangle$, $\langle H\rangle$ and $\langle W\rangle$, obtained in the first analysis step, are used as input parameters to calculate the line profiles. Based on comparison with the experimental line profiles, the values for $\langle D\rangle$, $\langle H\rangle$ and $\langle W\rangle$ are optimized to reproduce well the positions of the intensity maxima and minima in the profiles. These optimized values are reported in the main text. As an example, Supplementary Fig. 3 shows the experimental line profiles and calculated line profiles with optimized values for $\langle D\rangle$, $\langle H\rangle$ and $\langle W\rangle$ for Pt nanoparticles grown with the O_2-based process and Pt loading of ~115 atoms nm^{-2}.

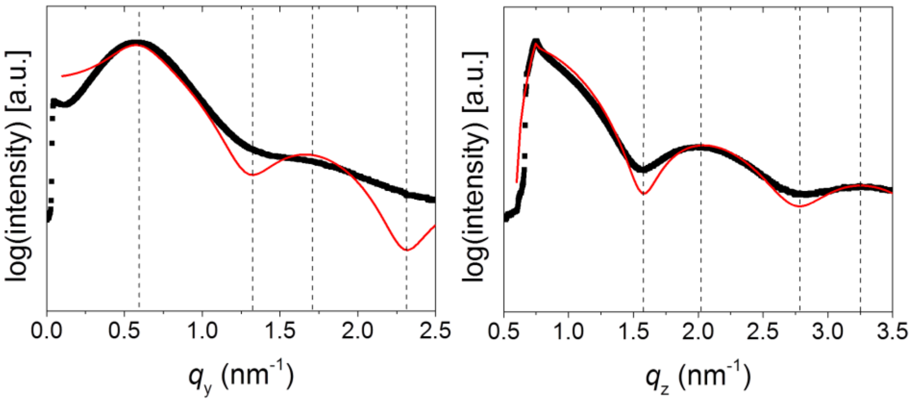

Morphological input parameters for the calculation in nm

⟨H⟩	⟨W⟩	⟨D⟩	ω
5.38	6.60	8.44	3.38

Supplementary Figure 3 | Extracting average morphological parameters from GISAXS line profiles. Experimental (black data points) and calculated (red curves) 1D horizontal (left graph, q_z = 0.722 nm^{-1}) and vertical (right graph, q_y = 0.59 nm^{-1}) line profiles. The vertical dashed lines indicate the good agreement in positions of the minima and maxima in both profiles. The table includes the input parameters that were used for the calculations. For form factor calculation, a mixture of 50% full spheroids and 50% hemispheroids was used.

To demonstrate how sensitive GISAXS is to changes in the parameters $\langle H \rangle$ and $\langle W \rangle$, Supplementary Fig. 4 illustrates the effect of systematic Ångstrom-level changes on the simulated line profiles. The simulations show that changes in the particle height of 2 Å can easily be distinguished by their change in oscillation period in the vertical line profile ((a), right graph). Similarly, a 2 Å deviation in the particle radius (4 Å in particle width) is shown to have a noticeable effect on the horizontal line profile ((b), left graph). In both cases, also the corresponding horizontal/vertical line profile has changed, though to a lesser extent.

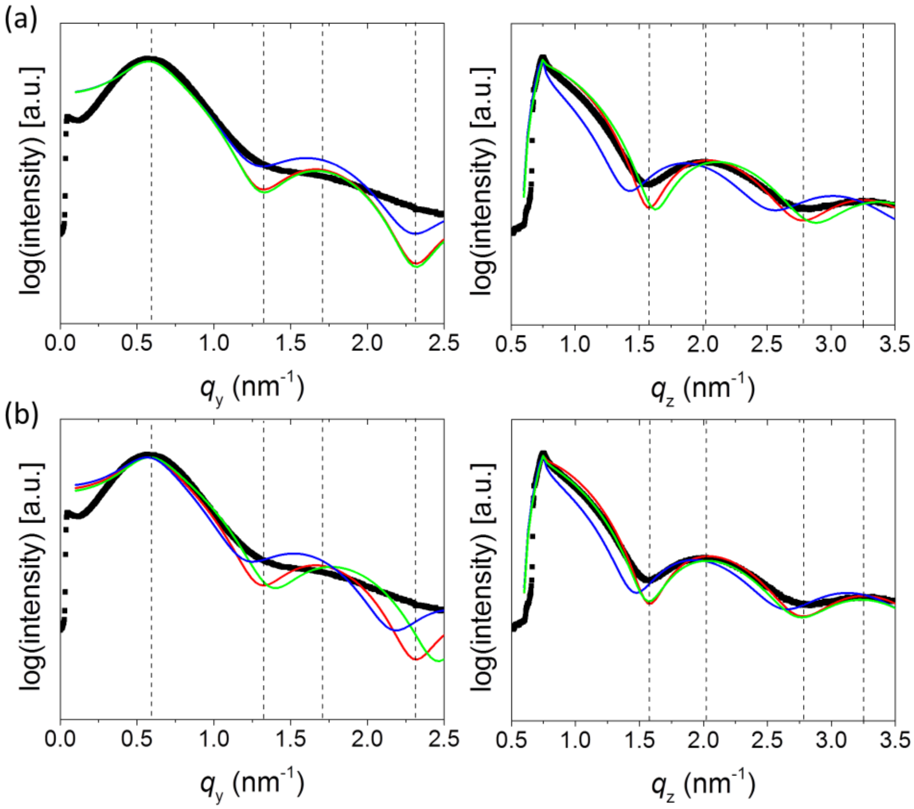

Morphological input parameters for the calculation in nm

⟨H⟩	⟨W⟩	⟨D⟩	ω
5.28	6.60	8.44	3.38
5.38	6.60	8.44	3.38
5.58	6.60	8.44	3.38

Morphological input parameters for the calculation in nm

⟨H⟩	⟨W⟩	⟨D⟩	ω
5.38	6.20	8.44	3.38
5.38	6.60	8.44	3.38
5.38	7.00	8.44	3.38

Supplementary Figure 4 | Sensitivity of GISAXS to changes in the average particle sizes. The sensitivity of GISAXS to particle height (a) and particle width (b) variations: experimental (black data points) and calculated (green, red and blue curves) 1D horizontal (left graph, q_z = 0.722 nm^{-1}) and vertical (right graph, q_y = 0.59 nm^{-1}) line profiles. The table includes the input parameters that were used for the calculations. For form factor calculation, a mixture of 50% full spheroids and 50% hemispheroids was used.

Step 3

The final step aims to validate the analysis approach that was used in steps 1 and 2 to extract the average particle height $\langle H \rangle$, particle width $\langle W \rangle$ and center-to-center particle distance $\langle D \rangle$ by simulating the complete 2D pattern using the derived values for $\langle D \rangle$, $\langle H \rangle$ and $\langle W \rangle$ as input parameters, and comparing it to the experimental one. To improve the agreement between simulation and experiment, the model that was used in step 2 to calculate the 1D line profiles is extended to account for the distribution in particle sizes. For the sake of simplicity, the particle height and width distributions are chosen to be coupled, in the sense that a distribution of particle radii at constant height/radius ratio implies also a distribution of particle heights. A lognormal distribution is assumed for the particle radius R, based on precedence in the literature[7-11]:

$$p(R) = \frac{1}{\sqrt{2\pi}\, R \ln(\sigma_R)} \exp\left(-\frac{1}{2}\left(\frac{\ln(R/\langle R \rangle)}{\ln(\sigma_R)}\right)^2\right) \qquad (2)$$

with σ_R the dimensionless geometric standard deviation. The size distribution is kept equal for both types of particles in the model (full spheroids and hemispheroids). The calculations furthermore use the local monodisperse approximation (LMA) formalism, which is commonly used for polydispersed systems.[5] As an illustration, Supplementary Fig. 5 compares simulations with and without size distribution for the same sample as in Supplementary Figures 3 and 4. The obvious effect of the size distribution is smoothening of the 1D line profiles, leading to an improved agreement with the experimental data for a σ_R-value of 1.1. Since the aim of the complete 2D simulations is to validate the derived values for $\langle D \rangle$, $\langle H \rangle$ and $\langle W \rangle$ rather than to derive the exact width of the particle size distribution, the σ_R-parameter was not treated as a fitting parameter but is kept constant to 1.1 for all simulations performed in this study.

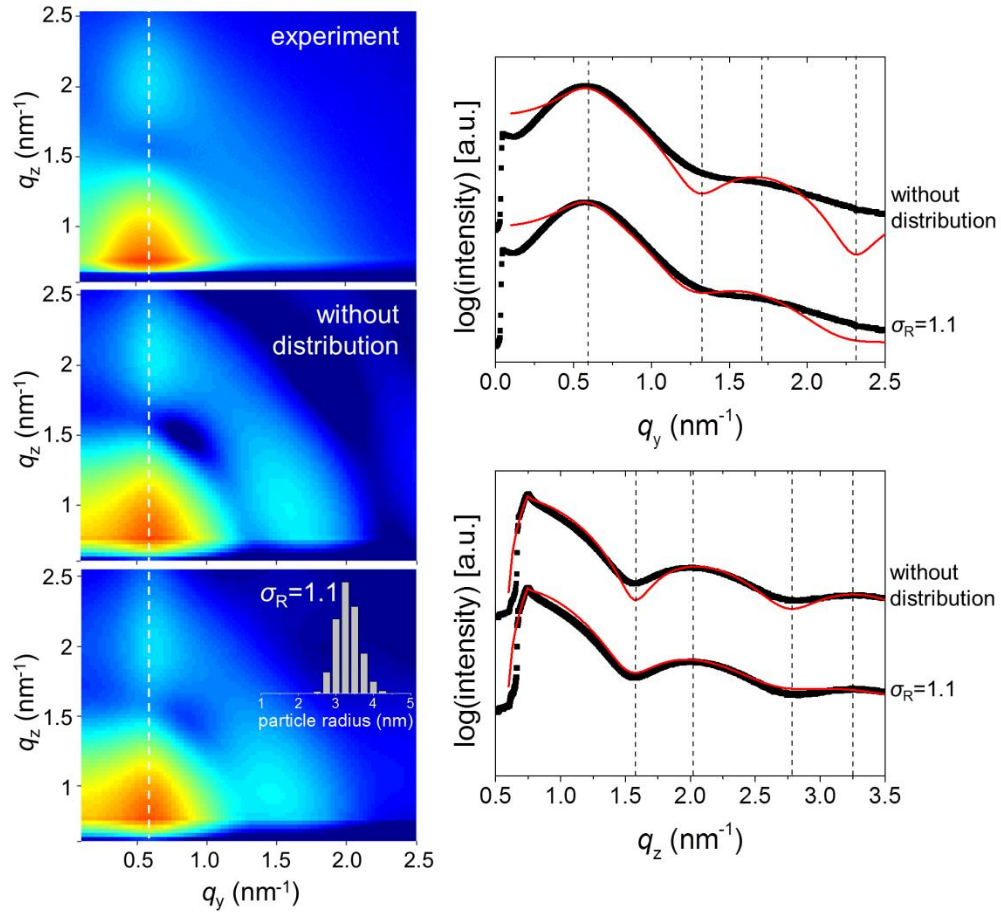

Supplementary Figure 5 | Effect of size dispersion on GISAXS simulations. Comparison between experimental and simulated GISAXS patterns calculated without and with coupled size distribution for the particle width and height: (left) 2D GISAXS patterns, (right) experimental (black data points) and calculated (red curves) 1D horizontal (top graph, q_z = 0.722 nm^{-1}) and vertical (bottom graph, q_y = 0.59 nm^{-1}) line profiles. The particle radius distribution is displayed in the top right corner of the respective simulated 2D GISAXS pattern. The other input parameters for the calculations are the same as those for the calculations in Supplementary Fig. 4. For form factor calculation, a mixture of 50% full spheroids and 50% hemispheroids was used.

Finally, to motivate our two-particle model for calculating the form factor, Supplementary Fig. 6 compares 2D patterns simulated for different spheroidal Pt nanoparticle geometries. In these simulations, the values for $\langle D\rangle$, $\langle H\rangle$ and $\langle W\rangle$, and those for ω and σ_R are kept constant, but the form factor is calculated for 100% full spheroids, 100% hemispheroids or a 50 to 50% mixture of both particle types. In the experimental 2D GISAXS pattern, one observes next to two clear

scattering peaks a less intense arc-like feature (marked by 1 in Supplementary Fig. 6) and a triangular scattering that emerges from the main scattering peak (marked by 2). Note that these scattering features are apparent in most of the experimental patterns recorded in this study. However, in the simulated scattering patterns for the one-particle models, one observes only one of these scattering features. In case of 100% full spheroids, the pattern is marked by a clear arc-like feature. In case of 100% hemispheroids, a clear scattering feature emerges from the main peak. By assuming a mixture of the two particle types for calculation of the form factor, the simultaneous appearance of the two scattering features, as observed in the experimental patterns, can be reproduced in the simulations. We believe that this mixture of two wetting conditions in the simulations corresponds to a real situation where the contact angle of the spheroidal Pt nanoparticles with the SiO_2 surface varies from particle to particle, as also suggested by the TEM tomography data in Figure 2 in the main text.

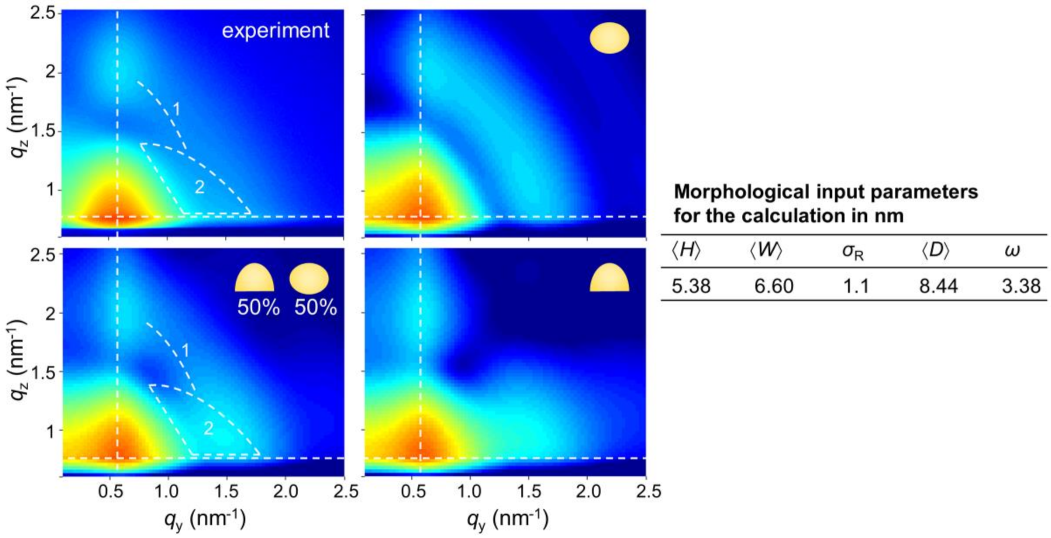

Morphological input parameters for the calculation in nm

$\langle H \rangle$	$\langle W \rangle$	σ_R	$\langle D \rangle$	ω
5.38	6.60	1.1	8.44	3.38

Supplementary Figure 6 | Effect of particle shape on GISAXS simulations. Comparison between experimental and simulated GISAXS patterns calculated for different spheroidal particle shapes. The particle shape assumed for calculation of the form factor is displayed in the top right corner of the respective simulated 2D GISAXS pattern. The table includes the input parameters that were used for the calculations.

By comparing the experimental GISAXS patterns for the O_2-based and N_2*-based ALD processes (Figure 1 in the main text), it is clear that the arc-like scattering feature is more

pronounced for the N_2*-based process, suggesting (on average) larger contact angles (larger dewetting) for the Pt nanoparticles deposited via this process. In the GISAXS simulations, this is taken into account by assuming a 75 to 25% ratio of full spheroids and hemispheroids to calculate the form factor.

It should be noted that a similar simulation approach was used before by Kaune et al. who reported a two-particle model consisting of parallelepipeds and spheroids to reproduce both the intensity distribution of the side peaks and the interconnecting streaks observed in experimental GISAXS patterns recorded for gold cluster growth on poly(N-vinylcarbazole).[12]

Supplementary Note 2: GISAXS simulation results

Supplementary Figures 7 to 11 show the simulated 2D GISAXS patterns for the experimental patterns shown in Figures 1a (left and right), 4, 5 and 6 in the main text, together with the 1D line profiles. The 2D patterns and 1D line profiles were calculated as explained in step 3 of the analysis strategy (Supplementary Note 1) and used values for $\langle D\rangle$, $\langle H\rangle$ and $\langle W\rangle$ that were obtained via steps 1 and 2 of the analysis procedure (Supplementary Note 1). The general input parameters for the calculations are listed in Supplementary Table 1, while the specific morphological input parameters are given in the tables included in Supplementary Figures 8 to 11. In general, a good agreement is found between the experimental data and the simulations. The main features such as position and relative intensity of the different maxima/minima are successfully reproduced, demonstrating the validity of our analysis approach to derive the average particle width, height and center-to-center distance. The discrepancy with the experimental images near $q_y = 0$ nm^{-1} arises from the interference function in the simulations showing a tail towards low q_y-values originating from larger, more widely spaced particles which are not present in the real samples.

For the N_2^*-based Pt ALD process (Supplementary Fig. 8), "fast analysis" according to step 1 of the analysis strategy (Supplementary Note 1) yields a quasi-constant value for $\langle D\rangle$ with the number of ALD cycles (less than 5% deviation). This result confirms that the fixed q_y-position of the main scattering lobe during the ALD process can be related to a fixed center-to-center distance $\langle D\rangle$, as is mentioned in the main text. In view of this result, the value for $\langle D\rangle$ is kept constant in the simulations for this type of ALD process.

Framework and beam parameters			
Framework	Diffuse scattering	Graded interface (GI)	# index slices for GI
DWBA	LMA	Yes	25
Beam wavelength:	λ (nm)	λ-distribution	
	0.0972	None	
Beam incidence angle:	α_i (˚)	α_i-distribution	
	0.500	None	
Substrate properties:	δ	β	
	3.0E-6	2.8E-6	
Particle properties:	δ	β	
	2.0E-5	2.5E-6	
**Grid parameters**			
q_y-range (nm^{-1})	q_z-range (nm^{-1})	Number of cells // q_y	Number of cells // q_z
0.1 – 2.5	0.6 – 3.5	80	100
**Particle parameters**			
	Shape	Probability	Flattening
Particle type 1:	spheroid	0.5 or 0.75	0.5*$\langle H\rangle/\langle R\rangle$ (= full spheroid)
Particle type 2:	spheroid	0.5 or 0.25	$\langle H\rangle/\langle R\rangle$ (= hemispheroid)
	Radius (nm)	Radius distribution	σ_R
Particle type 1 and 2:	$\langle R\rangle$	Lognormal	1.1
	Minimum radius (nm)	Maximum radius (nm)	# sampling points
Particle type 1 and 2:	1	7	25
	Height/radius	Height distribution	
Particle type 1 and 2:	$\langle H\rangle/\langle R\rangle$	Coupled	

Lattice parameters				
Particle distribution	Distance (nm)	Statistics	Disorder parameter (nm)	Cut-off (nm)
1D paracrystal	$\langle D\rangle$	Gaussian	Ω	100000

Supplementary Table 1 | Parameters used for the calculations in IsGISAXS.

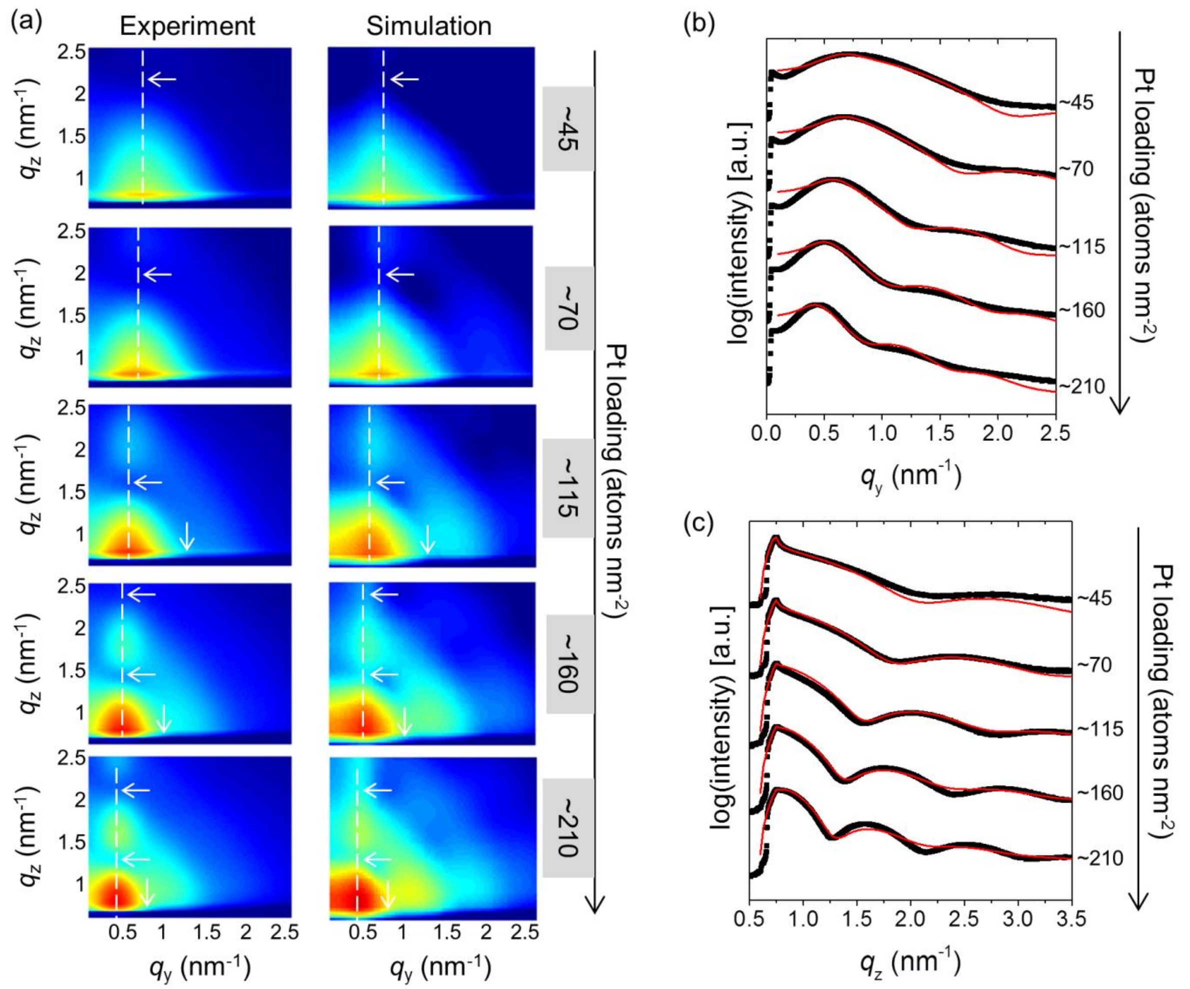

Morphological input parameters for the calculation (in nm)

Pt loading (atoms nm^{-2})	$\langle H \rangle$	$\langle W \rangle$	σ_R	$\langle D \rangle$	ω
~45	3.78	4.00	1.1	7.29	3.20
~70	4.30	5.00	1.1	7.43	2.97
~115	5.38	6.60	1.1	8.44	3.38
~160	6.20	7.60	1.1	9.65	3.86
~210	6.90	9.20	1.1	11.2	4.48

Supplementary Figure 7 | GISAXS simulations for O_2-based Pt ALD. (a) Comparison between experiment (left) and simulation (right) for a selection of 2D GISAXS images measured *in situ* during O_2-based Pt ALD. The dashed vertical lines indicate the q_y-position of the main scattering lobe. The horizontal/vertical arrows indicate the minima along the q_z/q_y-direction. (b,c) Corresponding experimental (black data points) and calculated (red curves) 1D horizontal (b) and vertical (c) line profiles. The horizontal line profiles are taken at the Si Yoneda position, i.e. q_z = 0.722 nm^{-1}. The vertical line profiles are taken at the q_y-position of maximum intensity, i.e. along the dashed vertical lines displayed in the 2D images in (a). The table includes the input parameters that were used for the calculations. For form factor calculation, a mixture of 50% full spheroids and 50% hemispheroids was used.

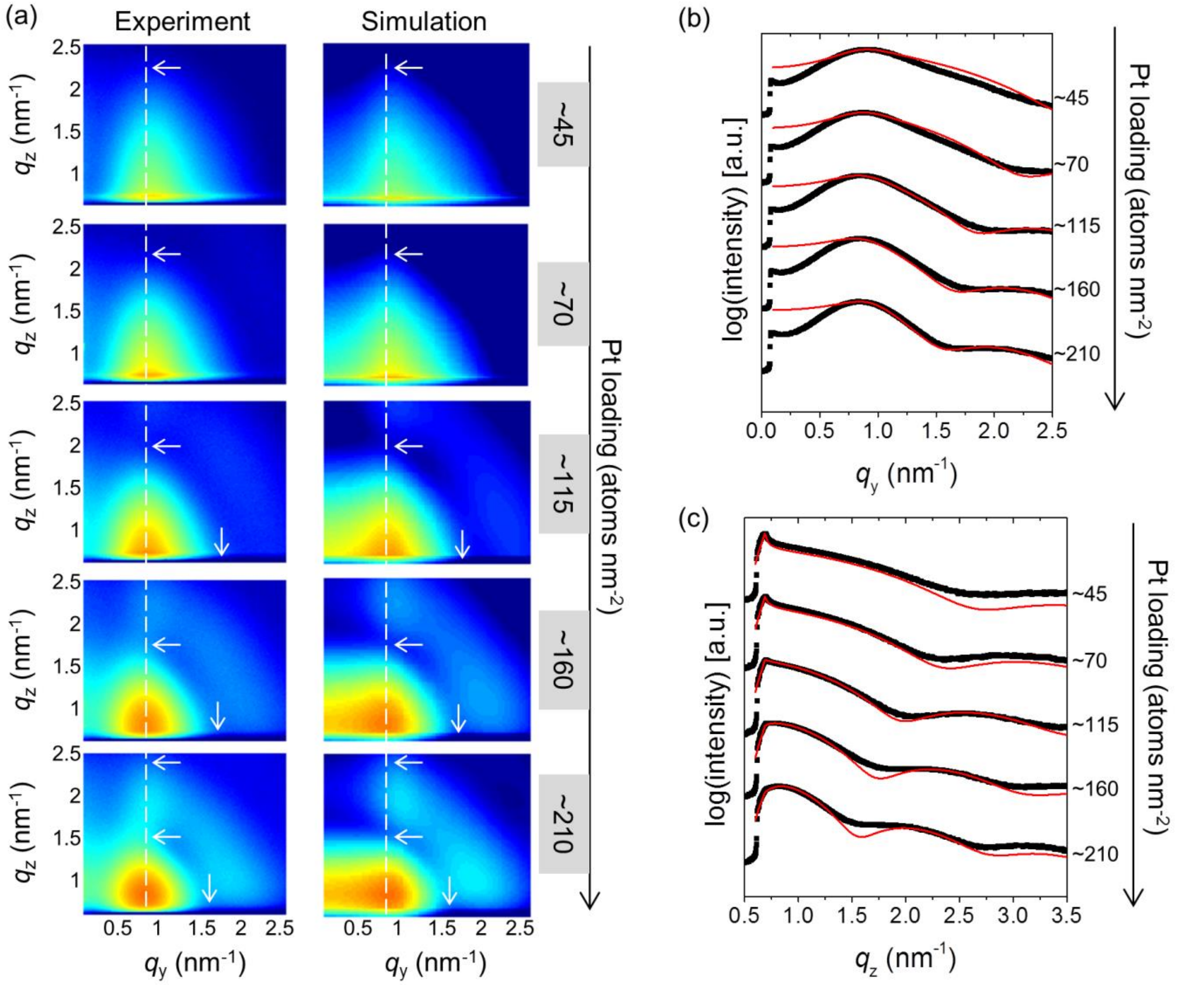

Morphological input parameters for the calculation (in nm)

Pt loading (atoms nm^{-2})	$\langle H \rangle$	$\langle W \rangle$	σ_R	$\langle D \rangle$	ω
~45	3.02	3.20	1.1	5.60	2.30
~70	3.40	3.80	1.1	5.60	2.30
~115	4.09	4.60	1.1	5.60	2.30
~160	4.74	5.10	1.1	5.60	2.30
~210	5.40	5.40	1.1	5.60	2.30

Supplementary Figure 8 | GISAXS simulations for N_2^*-based Pt ALD. (a) Comparison between experiment (left) and simulation (right) for a selection of 2D GISAXS images measured *in situ* during N_2^*-based Pt ALD. The dashed vertical lines indicate the q_y-position of the main scattering lobe. The horizontal/vertical arrows indicate the minima along the q_z/q_y-direction. (b,c) Corresponding experimental (black data points) and calculated (red curves) 1D horizontal (b) and vertical (c) line profiles. The horizontal line profiles are taken at the Si Yoneda position, i.e. $q_z = 0.722$ nm^{-1}. The vertical line profiles are taken at the q_y-position of maximum intensity, i.e. along the dashed vertical lines displayed in the 2D images in (a). The table includes the input parameters that were used for the calculations. For form factor calculation, a mixture of 75% full spheroids and 25% hemispheroids was used.

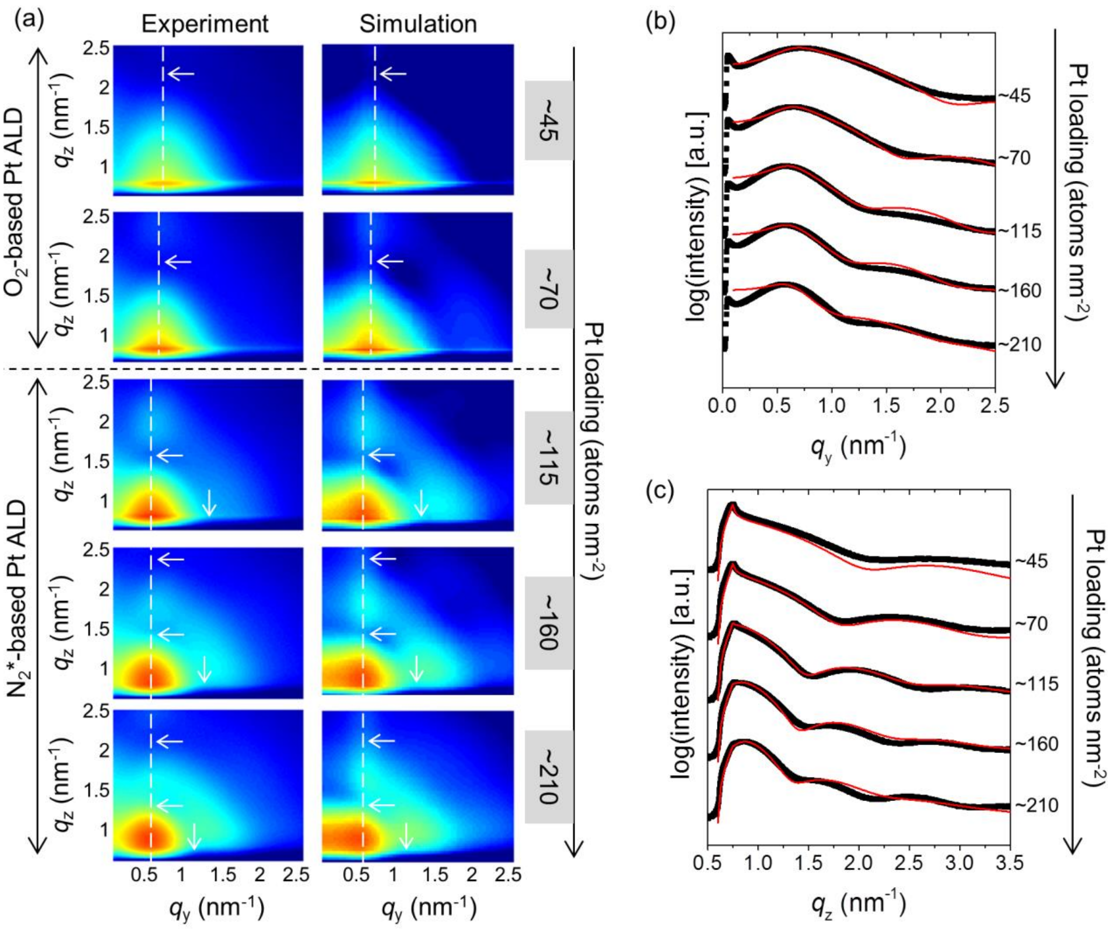

Morphological input parameters for the calculation (in nm)

Pt loading (atoms nm^{-2})	$\langle H \rangle$	$\langle W \rangle$	σ_R	$\langle D \rangle$	ω
~45	3.78	4.00	1.1	7.15	3.00
~70	4.38	5.00	1.1	7.70	3.08
~115	5.74	6.20	1.1	8.00	3.20
~160	6.29	6.80	1.1	8.00	3.20
~210	6.84	7.20	1.1	8.00	3.20

Supplementary Figure 9 | GISAXS simulations for the combined Pt ALD process. (a) Comparison between experiment (left) and simulation (right) for a selection of 2D GISAXS images measured *in situ* during the combined process using first O_2-based Pt ALD to tune the center-to-center particle distance followed by N_2^*-based Pt ALD to tune the particle size. The dashed vertical lines indicate the q_y-position of the main scattering lobe. The horizontal/vertical arrows indicate the minima along the q_z/q_y-direction. (b,c) Corresponding experimental (black data points) and calculated (red curves) 1D horizontal (b) and vertical (c) line profiles. The horizontal line profiles are taken at the Si Yoneda position, i.e. $q_z = 0.722\ nm^{-1}$. The vertical line profiles are taken at the q_y-position of maximum intensity, i.e. along the dashed vertical lines displayed in the 2D images in (a). The table includes the input parameters that were used for the calculations. For form factor calculation, a mixture of 50% full spheroids and 50% hemispheroids was used.

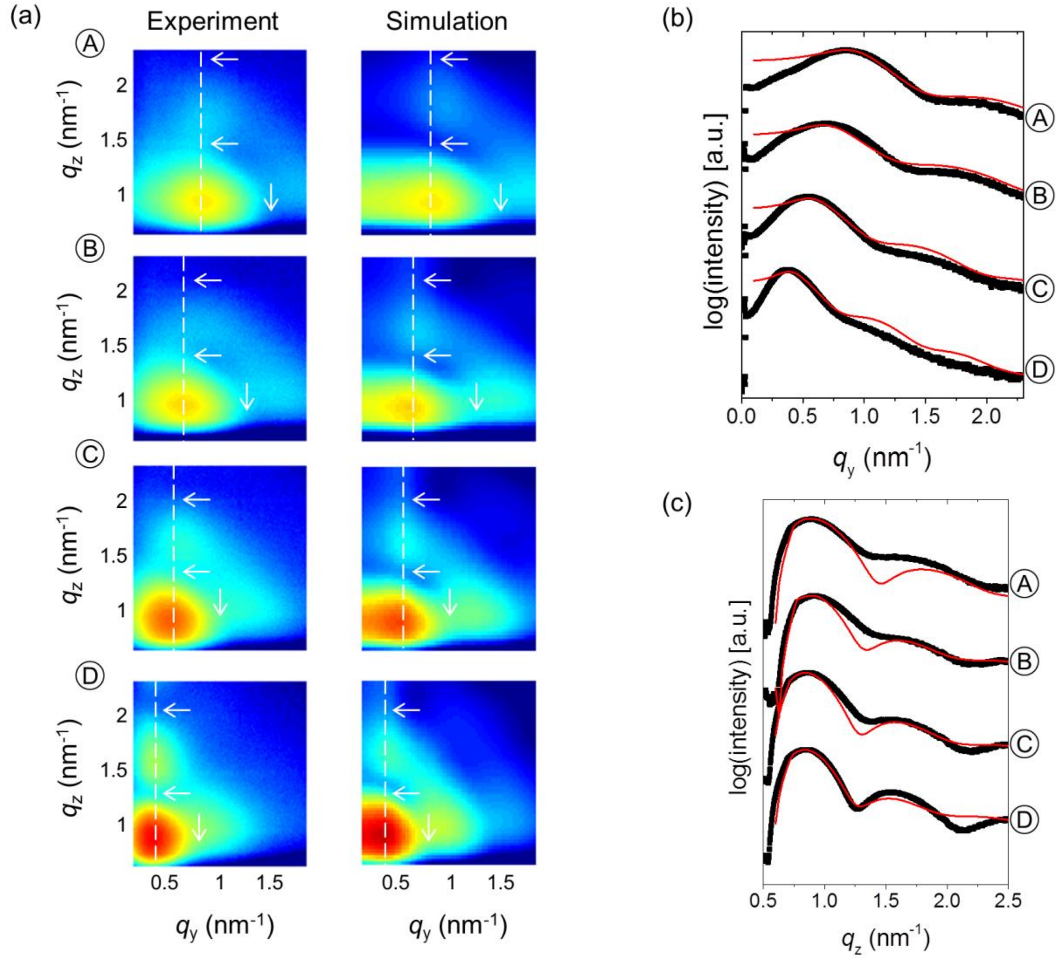

Morphological input parameters for the calculation (in nm)

Pt loading (atoms nm^{-2})		$\langle H\rangle$	$\langle W\rangle$	σ_R	$\langle D\rangle$	ω
Ⓐ	~210	6.21	5.40	1.1	5.60	2.00
Ⓑ	~190	7.04	6.20	1.1	7.10	2.90
Ⓒ	~200	7.22	7.80	1.1	8.70	3.50
Ⓓ	~210	7.28	10.4	1.1	10.8	4.80

Supplementary Figure 10 | GISAXS simulations for the "coverage tuning samples". (a) Tuning the particle coverage by combining 0 (sample A), 20 (sample B), 30 (sample C) and 40 (sample D) O_2-based Pt ALD cycles with 60 (sample A), 40 (sample B), 30 (sample C) and 20 (sample D) N_2^*-based Pt ALD cycles: experimental and simulated 2D GISAXS images. The dashed vertical lines in the GISAXS patterns indicate the q_y-position of the main scattering lobe. The horizontal/vertical arrows indicate the minima along the q_z/q_y-direction. (b,c) Corresponding experimental (black data points) and calculated (red curves) 1D horizontal (b) and vertical (c) line profiles. The horizontal line profiles are taken at the Si Yoneda position, i.e. $q_z = 0.722$ nm^{-1}. The vertical line profiles are taken at the q_y-position of maximum intensity, i.e. along the dashed vertical lines displayed in the 2D images in (a). The table includes the input parameters that were used for the calculations. For form factor calculation, a mixture of 50% full spheroids and 50% hemispheroids was used.

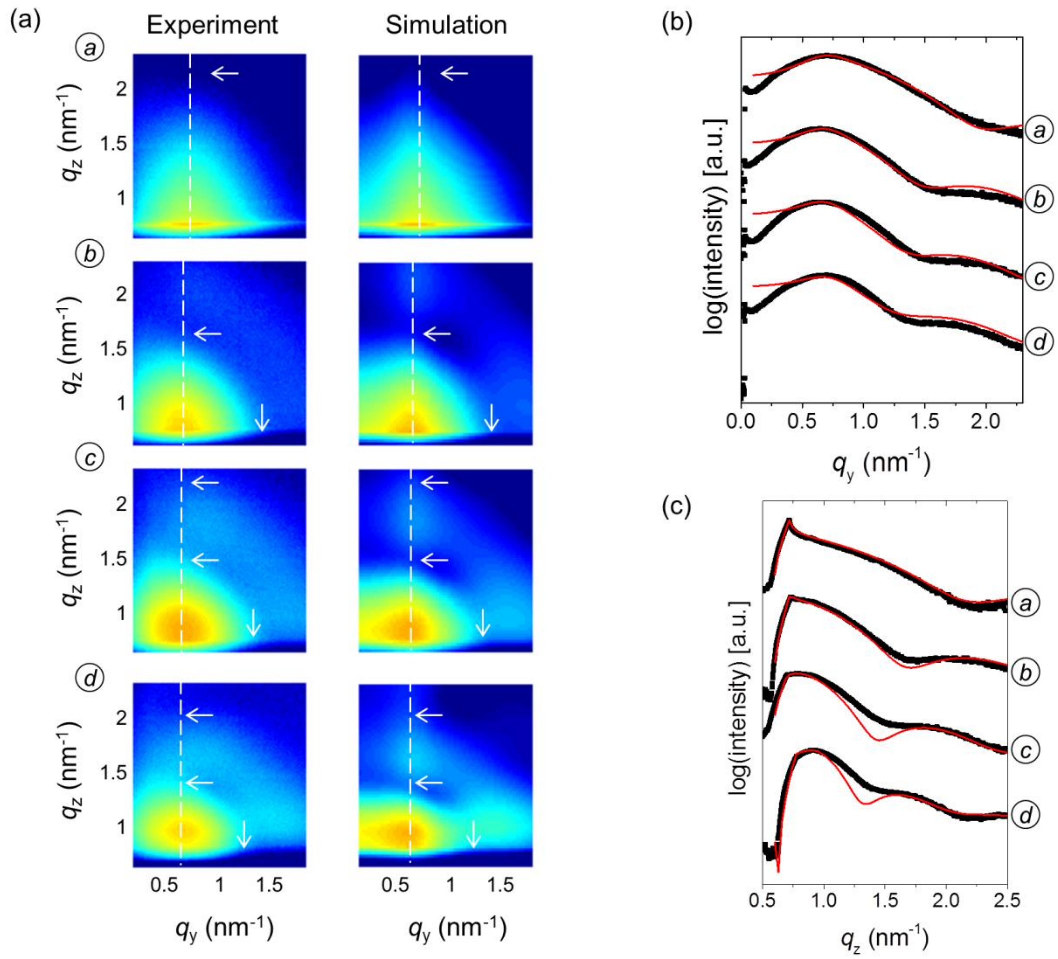

Morphological input parameters for the calculation (in nm)

Pt loading (atoms nm^{-2})		⟨*H*⟩	⟨*W*⟩	σ_R	⟨*D*⟩	ω
a	~35	3.63	4.40	1.1	7.10	2.90
b	~105	4.90	5.60	1.1	7.10	2.90
c	~135	6.00	6.00	1.1	7.10	2.90
d	~190	7.04	6.20	1.1	7.10	2.90

Supplementary Figure 11 | GISAXS simulations for the "size tuning samples". (a) Tuning the particle size by combining 20 O_2-based Pt ALD cycles with 0 (sample *a*), 20 (sample *b*), 30 (sample *c*), and 40 (sample *d*) N_2^*-based Pt ALD cycles: experimental (left) and simulated (right) 2D GISAXS images. The dashed vertical lines indicate the q_y-position of the main scattering lobe. The horizontal/vertical arrows indicate the minima along the q_z/q_y-direction. (b,c) Corresponding experimental (black data points) and calculated (red curves) 1D horizontal (b) and vertical (c) line profiles. The horizontal line profiles are taken at the Si Yoneda position, i.e. $q_z = 0.722\ nm^{-1}$. The vertical line profiles are taken at the q_y-position of maximum intensity, i.e. along the dashed vertical lines displayed in the 2D images in (a). The table includes the input parameters that were used for the calculations. For form factor calculation, a mixture of 50% full spheroids and 50% hemispheroids was used.

Supplementary Note 3: GISAXS uncertainty estimation

To estimate the uncertainties of the obtained values for $\langle H\rangle$, $\delta\langle H\rangle$, experimental and simulated 1D vertical line profiles were compared and the sum of squared residuals (SSR) was calculated for varying values of $\langle H\rangle$ and fixed values of $\langle W\rangle$, $\sigma_R = 1.1$, $\langle D\rangle$ and $\omega = 0.4\cdot\langle D\rangle$ (i.e. the values extracted from the GISAXS analysis approach discussed in Supplementary Note 1). The relative SSR was defined as the ratio between the SSR obtained at a certain $\langle H\rangle$ value and the SSR obtained at the $\langle H\rangle$ value extracted from our GISAXS analysis. Supplementary Fig. 12a, middle shows the relative SSR for a range of values of $\langle H\rangle$ for growth stages corresponding to a Pt loading of ~155 atoms nm^{-2} with the O_2-based (squares) and N_2^*-based (circles) Pt ALD process, respectively. In both cases, a parabolic-type trend is observed and the extracted $\langle H\rangle$ value (red data point) is found near its minimum. Similar results were obtained for all $\langle H\rangle$ values plotted in Figure 3a, bottom in the main text. This confirms again the validity of our analysis approach to derive the average particle height. The uncertainties of the extracted $\langle H\rangle$ values were then estimated by determining the values of $\langle H\rangle$ for which the relative SSR increased to ca. 1.5 (green and blue data points). This increase in SSR yielded 1D vertical line profiles which deviated from the optimized simulated line profile (resulting from the analysis strategy discussed in Supplementary Note 1) and the experimental profile, as illustrated in Supplementary Fig. 12a, right (O_2-based Pt ALD) and Supplementary Fig. 12a, left (N_2^*-based Pt ALD). The positions of the minima and maxima in the green and blue line profiles are clearly shifted with respect to the dashed vertical lines which indicate the extrema observed in the experimental 1D vertical line profiles. The uncertainties of the extracted $\langle H\rangle$ values were derived from the SSR analysis as indicated in Supplementary Fig. 12a, middle and were added as error bars ($\pm\delta\langle H\rangle$) in Figure 3 in the main text.

A similar strategy was used to estimate the uncertainties of the obtained values for $\langle W\rangle$ and $\langle D\rangle$ (Supplementary Figures 12b and 12c, respectively). In both cases, SSR values were calculated based on the comparison of experimental and simulated 1D horizontal line profiles. In these profiles, the value of $\langle D\rangle$ mainly influences the q_y-position of the main scattering peak (via the interference function), while the q_y-positions of the minima and second maximum mainly originate from the form factor and thus the value of $\langle W\rangle$. Therefore, calculation of the SSR values for varying values of $\langle D\rangle$ was done by limiting the q_y-range of the 1D horizontal line

profiles to the main scattering peak (Supplementary Fig. 12c). In contrast, calculation of the SSR values for varying values of $\langle W \rangle$ was done by excluding the q_y-range of the main scattering peak from the 1D horizontal line profiles (Supplementary Fig. 12b). For most of the $\langle W \rangle$ and $\langle D \rangle$ data points in Figure 3a, again a parabolic-type relation with a minimum near the extracted $\langle W \rangle$ and $\langle D \rangle$ values was found, allowing to extract the uncertainty of these values by evaluating the simulations with a relative SSR of ca. 1.5, as explained above for the uncertainty of $\langle H \rangle$ and as illustrated in Supplementary Figures 12b and 12c, respectively. For the N_2^*-based Pt ALD process and Pt loadings above ~160 atoms nm^{-2}, it was not possible to obtain a full parabola-type curve when varying $\langle D \rangle$ because of the constraint that $\langle D \rangle > \langle W \rangle$. In these cases, the uncertainty was estimated by evaluating only one simulation with a relative SSR of ca. 1.5 and doubling the obtained offset in $\langle D \rangle$.

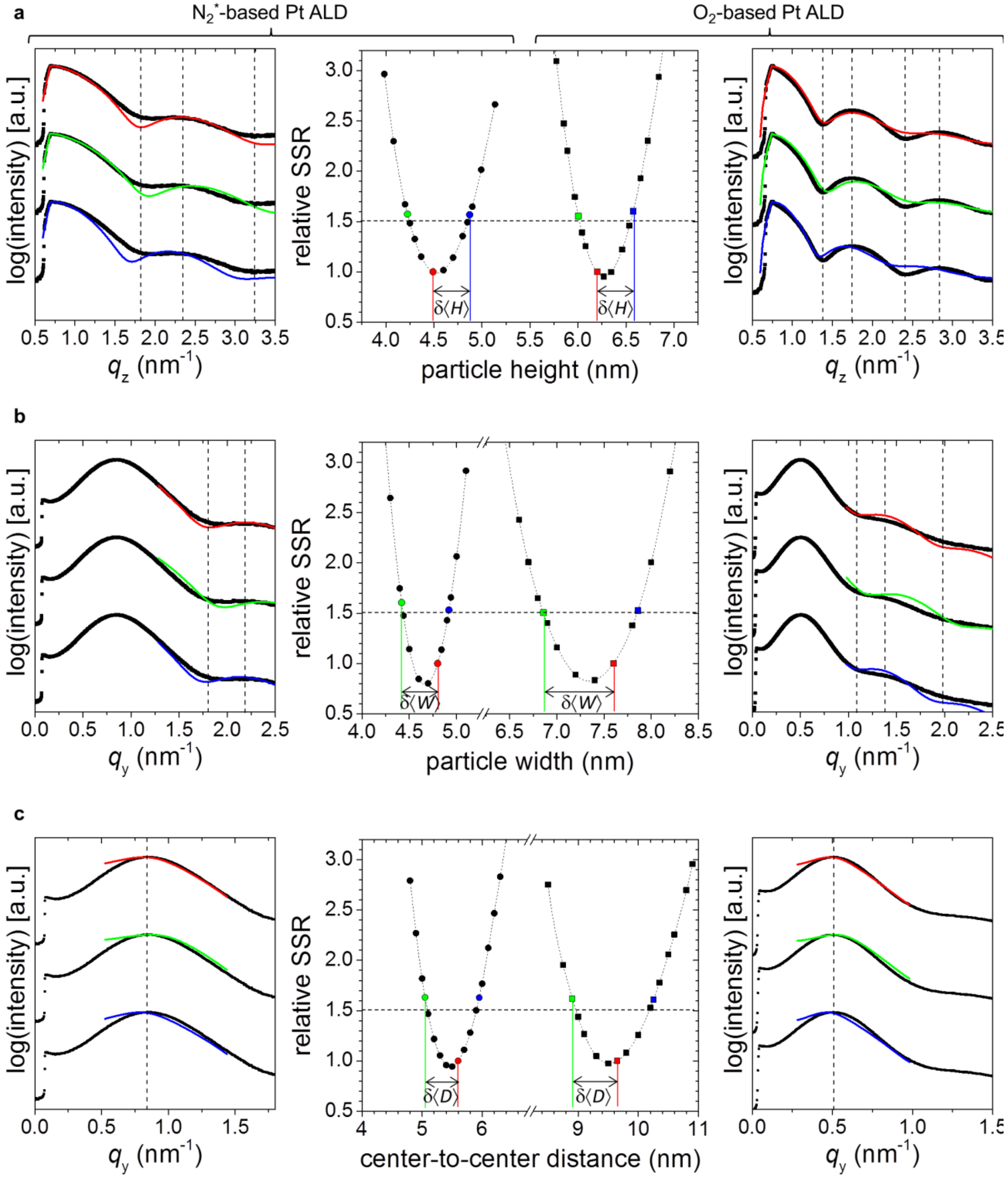

Supplementary Figure 12 | Uncertainty estimation of extracted morphological parameters. The graphs in the middle present the relative sum of squared residuals (SSR) against the particle height (a), particle width (b) and center-to-center distance (c) obtained for selected growth stages of the O_2-based Pt ALD process (squares) and N_2^*-based Pt ALD process (circles) corresponding to a Pt loading of ~155 atoms nm^{-2}. The graphs on the right and left display corresponding

experimental (black data points) and calculated (red/green/blue curves) 1D vertical (a) and horizontal (b,c) line profiles for the O_2-based Pt ALD process and N_2^*-based Pt ALD process, respectively. The horizontal line profiles are taken at the Si Yoneda position, i.e. $q_z = 0.722\ nm^{-1}$. The vertical line profiles are taken at the q_y-position of maximum intensity. The dashed vertical lines indicate positions of minima and maxima in the experimental line profiles. The red curves are calculated using the values for $\langle D\rangle$, $\langle H\rangle$ and $\langle W\rangle$ that were obtained via the analysis procedure described in Supplementary Note 1 (see red data points in the middle graphs). The green/blue curves correspond to an increase of the relative SSR to ca. 1.5 (see green/blue data points in the middle graphs). The estimated uncertainties $\delta\langle D\rangle$, $\delta\langle H\rangle$ and $\delta\langle W\rangle$ are indicated in the middle graphs and are added as error bars ($\pm\delta\langle D\rangle$, $\delta\langle H\rangle$ and $\delta\langle W\rangle$) in Figure 3 in the main text.

Supplementary Note 4: SEM/TEM analysis

As shown in Figure 8 in the main text, a good agreement is found between the average particle radius obtained from TEM analysis and the one derived from the GISAXS analysis. Supplementary Fig. 13 below presents additional analysis results for the SEM images included in Figure 6 of the main text, confirming again the agreement in average particle radius obtained from real-space electron microscopy measurements and reciprocal space GISAXS data. The black lines for samples A, B and C are fitted lognormal functions to the particle size distributions. The wide distribution observed for sample D is a consequence of the formation of wormlike structures when a large number of O_2-based ALD cycles is applied. For all lognormal fits, the value for the dimensionless geometric standard deviation σ_R is ~1.30. Similar fits to the size distributions obtained from TEM (Figure 8) yield a σ_R–value of ~1.25. Both of these values are larger than the value of 1.1 evaluated from GISAXS. However, for GISAXS simulations with a σ_R–value of 1.25 or 1.30, the scattering features are highly smoothed or damped, in disagreement with the experimental patterns. Similar differences in particle radius distribution obtained from TEM and GISAXS have been observed before for 1-10 nm Au nanoparticles embedded in a SiO_2 film and may be attributed to different sampling conditions.[13] For our SEM and TEM analyses, 300 to 1000 particles are measured from a small region of the sample (< 500 x 500 nm^2) while GISAXS probes a sample area of ca. 300 nm x 2 cm, averaging over an estimated 10^8 particles.

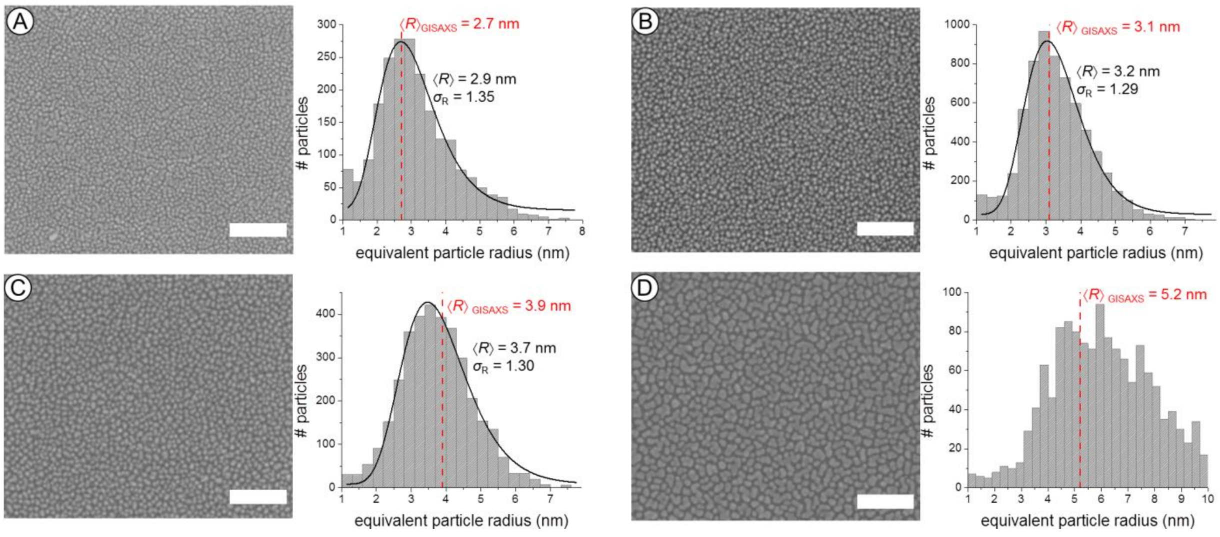

Supplementary Figure 13 | SEM characterization of the Pt nanoparticle size distribution. Tuning the particle coverage by combining 0 (sample A), 20 (sample B), 30 (sample C) and 40 (sample D) O_2-based Pt ALD cycles with 60 (sample A), 40 (sample B), 30 (sample C) and 20 (sample D) N_2^*-based Pt ALD cycles. SEM images with 100 nm scale bars and derived particle size distributions. The black lines are fitted lognormal functions to the data.

Supplementary Methods

Electrode preparation. Pt-ALD electrodes were fabricated on fluorine-doped tin oxide (FTO) coated glass slides (Sigma-Aldrich) with 10 Ω/sq surface resistivity. The FTO samples were cut in pieces of 2.5 by 1.5 cm. Prior to Pt deposition, surfaces were cleaned with acetone, isopropyl alcohol and deionized water. Subsequently ALD of Pt nanoparticles was performed using the O_2-based and N_2^*-based ALD processes. A conducting wire (Conrad, 0.14 mm^2) was attached on the surface of the substrate using silver paint (RS Components) and the contact was covered with epoxy resin (Loctite M-121MP Hysol, Henkel).

Electrochemical methods. Current-voltage curves were recorded at ambient temperature using a VersaSTAT 4 potentiostat (Princeton Applied Research) in a one-compartment electrochemical cell with electrolyte content of approximately 600 ml. A platinum coil (Bio-Logic) and an Ag/AgCl (3M KCl saturated with AgCl, Radiometer Analytical) served as counter and reference electrode, respectively. The H_2SO_4 electrolyte (Fisher Scientific, 95%) was diluted at 0.5 M concentration (pH 0.3) using Milli-Q water (18 MΩ cm). To investigate the catalyst activity, cyclic voltammetry measurements were performed after a 9 h 20 min stability measurement at *ca*. 7 mA cm^{-2}. Scan rate was set at 2 mV s^{-1}. During measurements, H_2 was purged through the solution and the solution was stirred with a magnetic stirring bar. Reported data were corrected for the uncompensated resistance (R_u) and current densities were normalized to the geometric surface area. R_u was determined by performing potentiostatic impedance spectroscopy in a frequency region between 1 Hz and 1 MHz. R_u was extracted from the data by taking the real part of the impedance in the high frequency region where the phase angle was zero. R_u varied between 9 and 12 Ω. Geometric areas were determined by making a photograph of the electrode and using the software *Image J* to calculate the area, and values of 0.15 and 0.21 cm^2 were obtained for the O_2-based and N_2^*-based Pt-ALD electrodes, respectively. The measured potentials were converted to the potentials against the reversible hydrogen electrode (RHE). Turnover frequencies (TOF) were calculated using the following equation:

$$TOF = \frac{I}{2\,F\,n} \qquad \text{(S.3)}$$

with I the current (A), F the Faraday constant (C mol^{-1}) and n the number of moles of the deposited Pt, which was determined from XRF results.

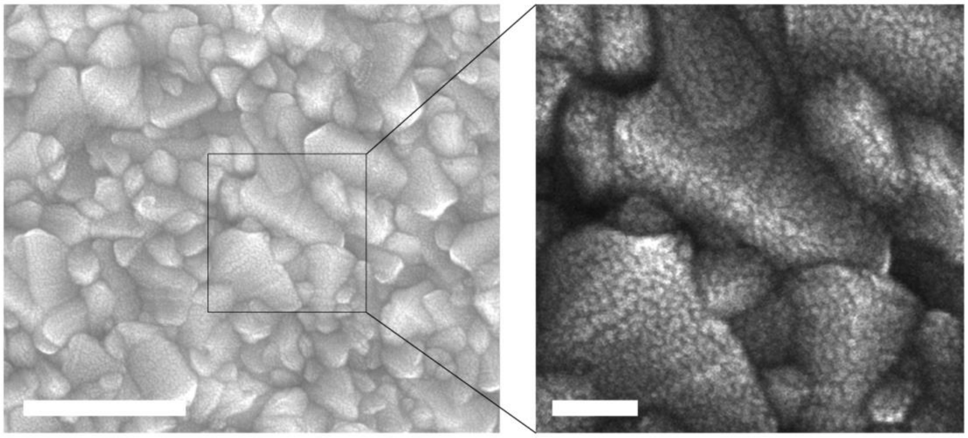

Supplementary Figure 14 | Morphological characterization of Pt nanostructures on FTO. SEM images of worm-like Pt nanostructures deposited with the O_2-based Pt ALD process on a FTO coated glass slide. The sample contains *ca.* 5 µg of Pt per cm^2 of glass substrate. The scale bars indicate 500 nm (left) and 100 nm (right). In the right image, the contrast is enhanced for an improved visibility of the Pt nanostructures.

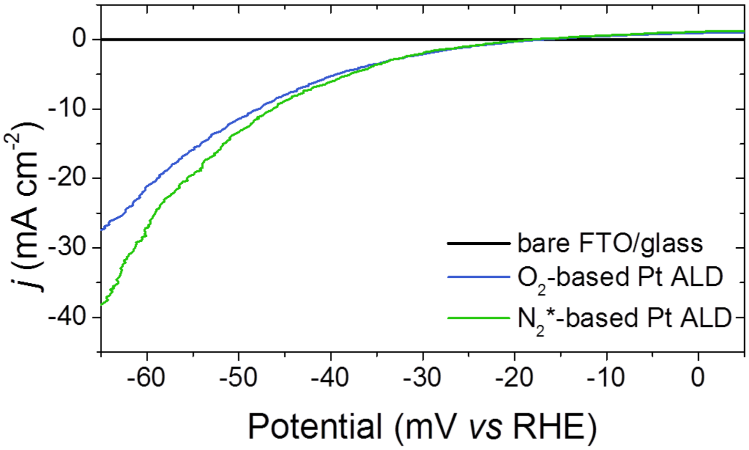

Supplementary Figure 15 | Evaluation of Pt nanoparticles in the HER of water electrolysis. Cyclic voltammograms of Pt nanoparticles deposited with the O_2-based and N_2^*-based ALD processes on FTO coated glass slides. Scan rate was 2 mV s^{-1}. Both samples contain *ca.* 3.5 µg of Pt per cm^2 of glass substrate. The black curve was measured with a bare FTO-coated glass substrate. The current density is calculated based on the geometric surface area. Raw data were smoothed with a 20-point moving average. Forward and backward scans of a single cycle were averaged to obtain the plot. Turnover frequencies at 50 mV overpotential were calculated to be 4.1 s^{-1} and 3.3 s^{-1} for the N_2^*- and O_2-based sample, respectively.

Supplementary References

(1) Schwartzkopf, M., Buffet, A., Körstgens, V., Metwalli, E., Schlage, K., Benecke, G., Perlich, J., Rawolle, M., Rothkirch, A., Heidmann, B., Herzog, G., Müller-Buschbaum, P., Röhlsberger, R., Gehrke, R., Stribeck, N. & Roth, S. V. From atoms to layers: in situ gold cluster growth kinetics during sputter deposition. *Nanoscale* **5**, 5053-5062 (2013).

(2) Santoro, G., Yu, S., Schwartzkopf, M., Zhang, P., Vayalil, S. K., Risch, J. F. H., Rübhausen, M. A., Hernández, M., Domingo, C. & Roth, S. V. Silver substrates for surface enhanced Raman scattering: correlation between nanostructure and Raman scattering enhancement. *Appl. Phys. Lett.* **104**, 243107 (2014).

(3) Schwartzkopf, M., Santoro, G., Brett, C. J., Rothkirch, A., Polonskyi, O., Hinz, A., Metwalli, E., Yao, Y., Strunskus, T., Faupel, F., Müller-Buschbaum, P. & Roth, S. V. Real-time monitoring of morphology and optical properties during sputter deposition for tailoring metal−polymer interfaces. *ACS Appl. Mater. Interfaces* **7**, 13547−13556 (2015).

(4) Lazzari, R. IsGISAXS: a program for grazing-incidence small-angle X-ray scattering analysis of supported islands. *J. Appl. Crystallogr.* **35**, 406-421 (2002).

(5) Renaud, G., Lazzari, R. & Leroy, F. Probing surface and interface morphology with grazing incidence small angle x-ray scattering. *Surf. Sci. Rep.* **64**, 255-380 (2009).

(6) Lazzari, R., Leroy, F. & Renaud, G. Grazing-incidence small-angle x-ray scattering from dense packing of islands on surfaces: development of distorted wave Born approximation and correlation between particle sizes and spacing. *Phys. Rev. B* **76**, 125411 (2007).

(7) Blackman, J. A., Evans, B. L. & Maaroof, A. I. Analysis of island-size distributions in ultrathin metallic-films. *Phys. Rev. B* **49**, 13863-13873 (1994).

(8) Söderlund, J., Kiss, L. B., Niklasson, G. A. & Granqvist, C. G. Lognormal size distributions in particle growth processes without coagulation. *Phys. Rev. Lett.* **80**, 2386-2388 (1998).

(9) Kiss, L. B., Söderlund, J., Niklasson, G. A. & Granqvist, C. G. New approach to the origin of lognormal size distributions of nanoparticles. *Nanotechnology* **10**, 25-28 (1999).

(10) Meshot, E. R., Verploegen, E., Bedewy, M., Tawfick, S., Woll, A. R., Green, K. S., Hromalik, M., Koerner, L. J., Philipp, H. T., Tate, M. W., Gruner, S. M. & Hart, A. J. High-speed in situ X-ray scattering of carbon nanotube film nucleation and self-organization *ACS Nano* **6**, 5091-5101 (2012).

(11) Qadri, M. U., Diaz, A. F. D., Cittadini, M., Martucci, A., Pujol, M. C., Ferré-Borrull, J., Llobet, E., Aguiló, M. & Díaz, F. Effect of Pt Nanoparticles on the optical gas sensing properties of WO_3 thin films. *Sensors* **14**, 11427-11443 (2014).

(12) Kaune, G., Ruderer, M. A., Metwalli, E., Wang, W., Couet, S., Schlage, K., Röhlsberger, R., Roth, S. V. & Müller-Buschbaum, P. In situ GISAXS study of gold film growth on conducting polymer films. *ACS Appl. Mater. Interfaces* **1**, 353-362 (2009).

(13) Sanchez, D. F., Marmitt, G., Marin, C., Baptista, D. L., Azevedo, G. D., Grande, P. L. & Fichtner, P. F. P. New approach for structural characterization of planar sets of nanoparticles embedded into a solid matrix. *Sci. Rep.* **3**, 3414 (2013).
