# Supplementary material for: Independent tuning of size and coverage of supported Pt nanoparticles using atomic layer deposition
Source: Nat Commun. 2017 Oct 20;8:1074. doi: 10.1038/s41467-017-01140-z (PMC5651928; doi:10.1038/s41467-017-01140-z)
Supplement: Supplementary file 3 — Description of Additional Supplementary Information [file 41467_2017_1140_MOESM3_ESM.pdf]

Description of Additional Supplementary Files

File Name: Supplementary Movie 1
Description: Electron tomography characterization of the Pt nanoparticle shape: animated 3D volume rendering of Pt nanoparticles synthesized with the O_2-based ALD process (Pt loading ~45 atoms nm^{-2}).
